# Supplementary material for: Reversible switching of the environment-protected quantum spin Hall insulator bismuthene at the graphene/SiC interface
Source: Nat Commun. 2025 Jul 4;16:6171. doi: 10.1038/s41467-025-60440-x (PMC12227741; doi:10.1038/s41467-025-60440-x)
Supplement: Supplementary file 1 — Supplementary Information [file 41467_2025_60440_MOESM1_ESM.pdf]

Supplementary Information for Reversible Switching of the Environment-Protected Quantum Spin Hall Insulator Bismuthene at the Graphene/SiC Interface

Niclas Tilgner[1,2], Susanne Wolff[1,2], Serguei Soubatch[3,4], Tien-Lin Lee[5], Andres David Peña Unigarro[1], Sibylle Gemming[1], F. Stefan Tautz[3,4,6], Thomas Seyller[1,2], Christian Kumpf[3,4,6,‡], Fabian Göhler[1,2,†], and Philip Schädlich[1,2,*]

[1]Institute of Physics, Chemnitz University of Technology, 09126 Chemnitz, Germany
[2]Center for Materials, Architectures and Integration of Nanomembranes (MAIN), 09126 Chemnitz, Germany
[3]Peter Grünberg Institut (PGI-3), Forschungszentrum Jülich, 52425 Jülich, Germany
[4]Jülich Aachen Research Alliance (JARA), Fundamentals of Future Information Technology, 52425 Jülich, Germany
[5]Diamond Light Source Ltd., Harwell Science and Innovation Campus, Didcot, Oxfordshire, OX11 0DE, United Kingdom
[6]Experimentalphysik IV A, RWTH Aachen University, 52074 Aachen, Germany
[‡]*Corresponding author E-Mail:* c.kumpf@fz-juelich.de
[†]*Corresponding author E-Mail:* fabian.goehler@physik.tu-chemnitz.de
[*]*Corresponding author E-Mail:* philip.schaedlich@physik.tu-chemnitz.de

May 21, 2025

1 Step structure of the 4H-SiC substrate

In Figure S.1(a) we show an atomic force microscopy (AFM) image of a graphene buffer layer sample on 4H-SiC prior to Bi intercalation. From left to right, a sequence of step edges can be seen, separating flat terraces that are about 0.3 µm wide. The height profile in (b), recorded along the black line shown in (a), indicates a typical step height of $\sim$ 0.5 nm, a value corresponding to the height of two SiC bilayers, that is half of the 4H-SiC unit cell. This allows for either S1/S1* or S2/S2* terminated terraces, but since the first is energetically unfavourable [1], we conclude that the vast majority of the surface is S2 and S2* terminated.

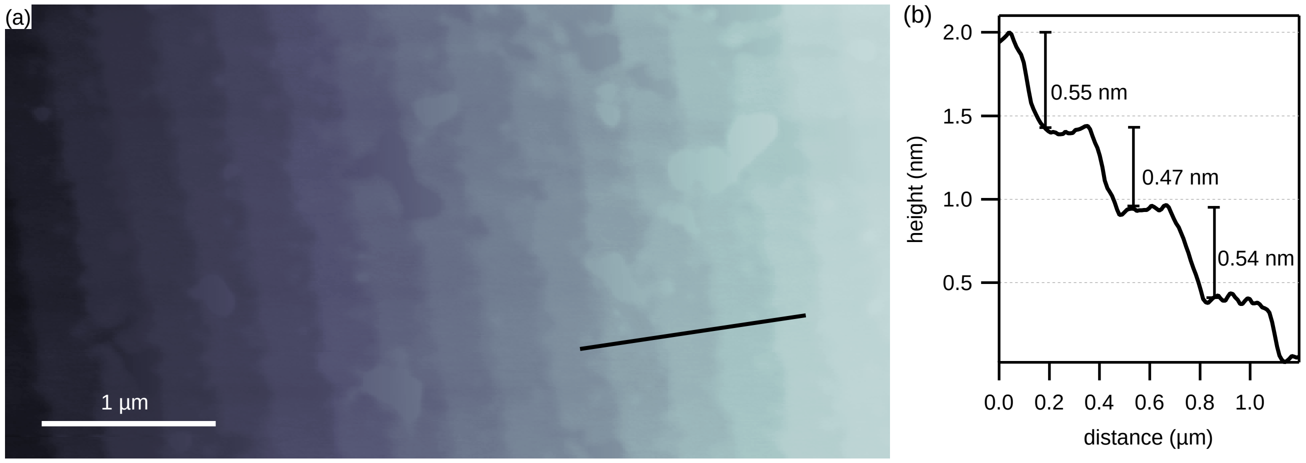

Figure S.1: (a) AFM image showing the topography of a buffer layer (zeroth layer graphene) on a 4H-SiC(0001) surface before Bi intercalation. (b) Height profile measured along the black line in (a).

2 Low-energy electron diffraction: I(V) analysis of the precursor and the bismuthene phase

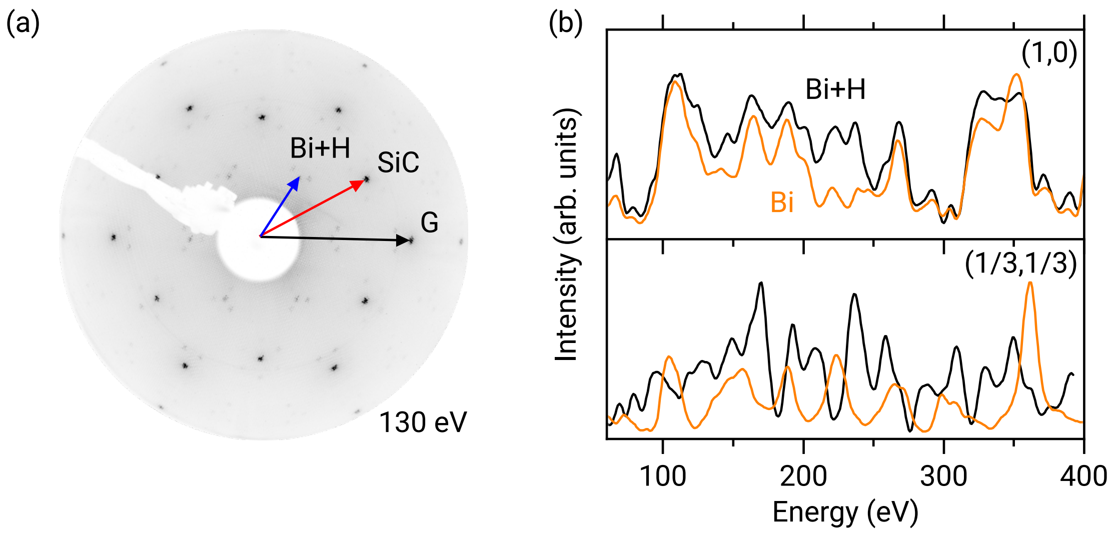

Figure S.2: (a) LEED pattern (E = 130 eV) with selected reciprocal lattice vectors of graphene (G), SiC, and bismuthene (Bi+H) highlighted. (b) LEED-IV spectra for selected beams before (Bi, orange) and after (Bi+H, black) the hydrogenation process.

Evidence for a significant structural rearrangement of the Bi layer upon hydrogenation can be obtained from low-energy electron diffraction. Figure S.2(a) shows the LEED pattern obtained for the bismuthene phase. At this electron energy, the pattern is very similar to that of the precursor phase (Figure 1a of the main text). In particular, the $(\sqrt{3}\times\sqrt{3})\mathrm{R}30^\circ$ superstructure associated with the Bi layer (blue arrows labeled Bi+H) is robust throughout the hydrogenation process. However, the LEED-IV spectra shown in Figure S.2(b) for one bulk reflection (the (10), red arrow in (a)) and one reflection from the Bi layer (the $(\frac{1}{3}\frac{1}{3})$, blue arrow) reveal clear differences in the intensities of the reflections for the two phases, in particular for the second reflection. We performed a Pendry analysis, known as a way to quantify the relevant differences in LEED-IV spectra [2]. For the SiC bulk spots we obtain $R_\mathrm{p} = 0.24$, for the first order diffraction spots of the $(\sqrt{3}\times\sqrt{3})\mathrm{R}30^\circ$ superstructure $R_\mathrm{p} = 1.03$. The large value obtained for the latter is a clear indication of fundamental structural changes in the interfacial structure, as associated with a change of the Bi adsorption site from T_4 to T_1.

3 Theoretical model and spin texture

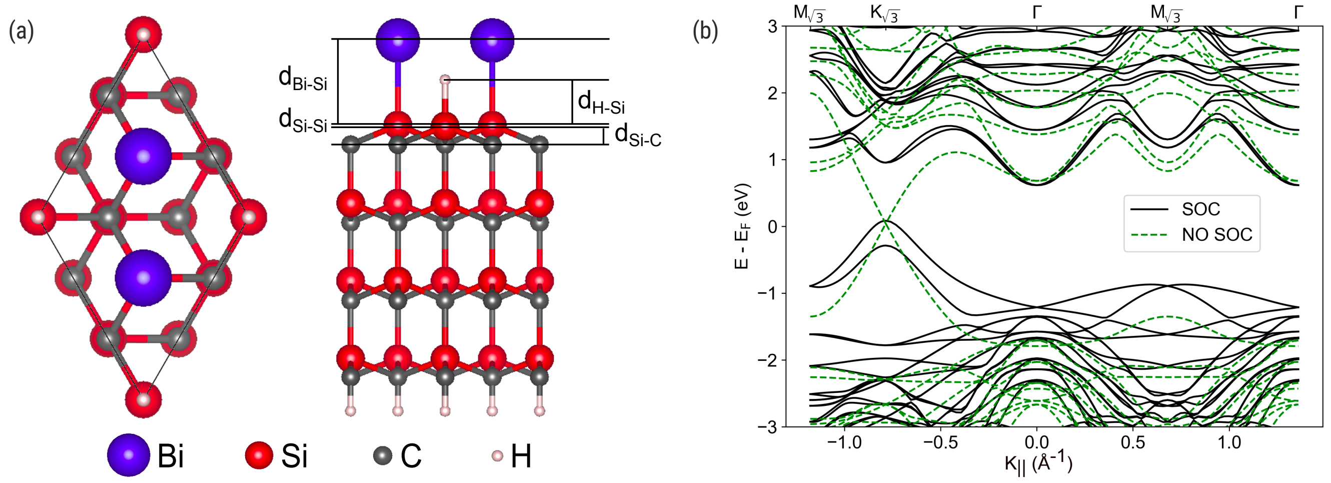

Figure S.3: (a) Atomic structure of the bismuthene phase on 4H-SiC after hydrogenation (left: top view, right: side view onto the (10$\bar{1}$0) plane of SiC). (b) Corresponding calculated band structure. Black solid lines and green dashed lines represent calculations with and without consideration of the spin-orbit coupling, respectively.

In Figure S.3 (a), a ball and stick model of bismuthene on SiC (after hydrogenation) is presented. In our calculations, the details of which can be found in the methods section of the main text, a lattice constant of 5.33 Å was used. After performing structural relaxation, the obtained distance between Bi and Si atoms was $d_{\text{Bi-Si}} = 2.75$ Å, which is in excellent agreement with the experimental value found by NIXSW imaging (2.74 Å, see the main text). For the hydrogen atom located on top of the top-most Si atom, a Si-H distance of 1.5 Å was obtained. Furthermore, the top-most Si plane is found slightly buckled ($d_{\text{Si-Si}} = 0.034$ Å) and a vertical separation between the Si and C atoms $d_{\text{Si-C}} = 0.64$ Å (0.606 Å for the Si atom saturated with H) was observed.

In Figure S.3 (b) the calculated electronic structure is displayed. Black solid lines and green dashed lines represent calculations with and without taking into account spin-orbit coupling (SOC), respectively. Note that a Dirac-like dispersion appears at the $K_{\sqrt{3}}$ point in the case that SOC was not included. Once SOC is included in the calculations, the two corresponding bands split with a gap of 0.9 eV and a global indirect band gap of 0.54 eV is obtained. Moreover, if SOC was included in the calculation, a Rashba-type spin splitting of 0.37 eV is found in the top valence band at the $K_{\sqrt{3}}$ point, as shown in Figure S.4.

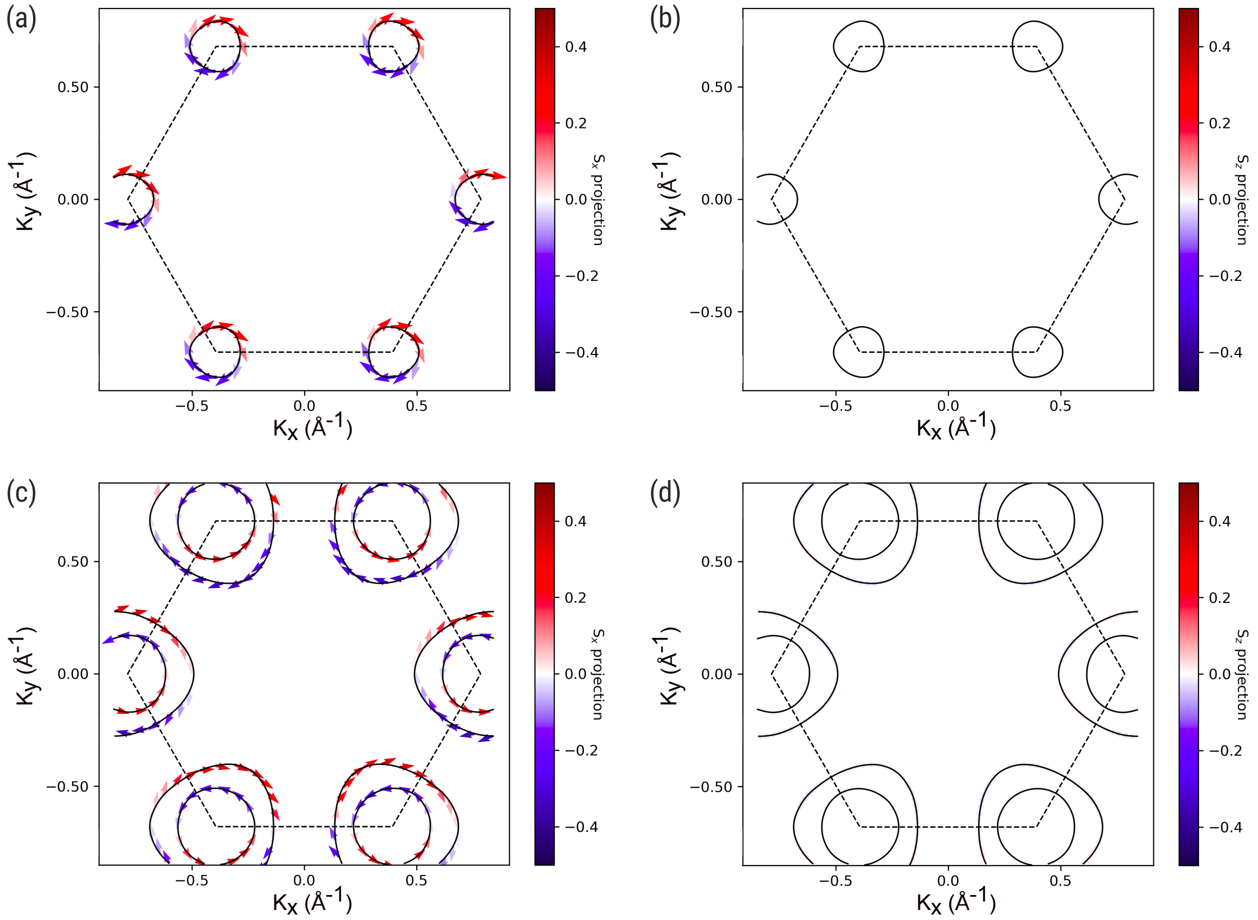

Figure S.4: Calculated spin texture for bismuthene (a,b) at E_{F} and (c,d) at $E_{\mathrm{B}} = 0.5$ eV. (a) and (c) represent an in-plane projections (S_x), (b and d) out-of-plane projections (S_z).

To confirm the Rashba splitting of the top valence band induced by the broken inversion symmetry of the bismuthene layer, 2D constant energy contours of the spin texture were calculated in the $k_x - k_y$ plane centered at the Γ point. Figure S.4 shows the projected S_x and S_z spin components on the constant energy contours at (a,b) E_{F} and (c,d) $E_{\mathrm{B}} = 0.5$ eV. In (a) we observe a radial spin polarization for the upper branch of the top valence band around the six $\mathrm{K}_{\sqrt{3}}$ points. The second branch, which is visible at $E_{\mathrm{B}} = 0.5$ eV (panel (c)), shows the opposite spin polarisation. In both cases, however, the out-of-plane S_z component is negligible, as seen in (b) and (d), indicating a complete in-plane spin polarization as it is characteristic of Rashba-type spin splitting.

4 X-ray photoelectron spectroscopy analysis of core levels

In Figures S.5(a)-(c) we show representative X-ray photoelectron spectra of the precursor phase and the bismuthene phase. Upon hydrogenation the shape of the Bi $4f$ peak changes from symmetric to asymmetric, as can be seen in panel (a): While the Bi $4f$ peak before hydrogenation (upper spectrum labelled Bi) can be described by a symmetric line profile, a similar fit after hydrogenation (lower spectrum, black data points labelled Bi+H) fails, as also indicated by the residuals of both fits shown in the inset. This change in spectral shape is in agreement with the transition from an insulating state in the initial precursor phase to a p-doped metallic state of the bismuthene seen in the ARPES measurements (see main text). The ratio of the (integrated) intensities of Bi $4f$ and the Si $2p$ is barely affected by hydrogenation, suggesting a stable overall coverage of Bi. In conjunction with the LEED diffraction patterns, this suggests a similar in-plane arrangement of the Bi atoms. Note that the Bi $4f$ signal does not change after exposing the sample to ambient conditions for 24 h (Figure S.5(a), bottom, red data points), see also Sec. 5.

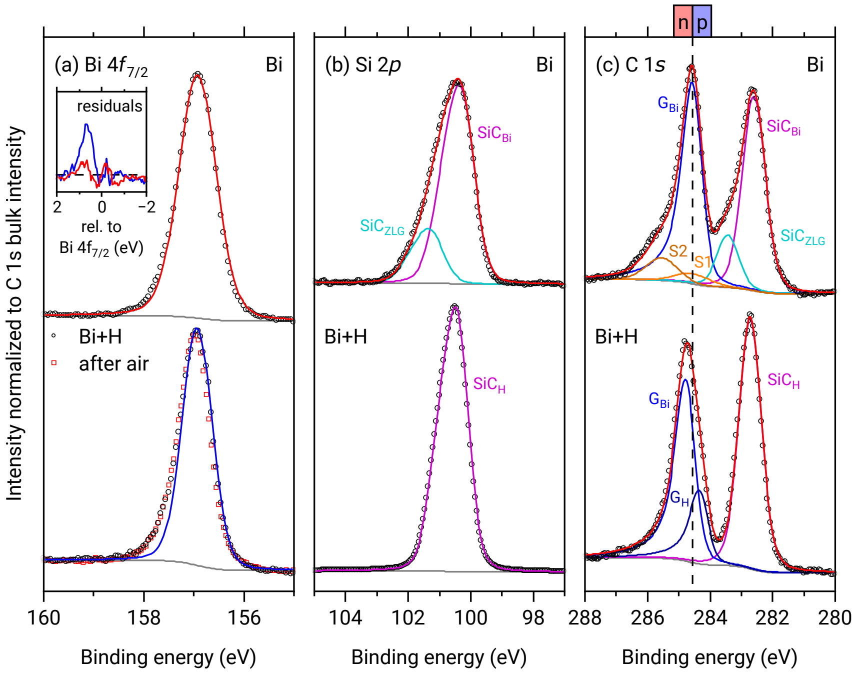

Figure S.5: (a-c) X-ray photoelectron spectra of the Bi $4f_{7/2}$, Si $2p$ and C $1s$ core levels, respectively, for the precursor (top) and the bismuthene phase (bottom). In (a), the two spectra (black data points) were fitted by symmetric line profiles (solid lines). The residuals shown in the inset indicate a change in the line profile upon hydrogenation. The spectrum plotted as red data points in the bottom of panel (a) was recorded after exposure of the sample to air. The Si $2p$ (b) and C $1s$ spectra (c) were fitted using multiple components, see text. The vertical, dashed line in (c) indicates the C $1s$ peak position for charge neutral graphene.

The Si $2p$ and C $1s$ spectra (see Figures S.5 (b) and (c)) have more complicated profiles due to more complex bonding environments of the involved atomic species. Before hydrogenation, the bulk contributions of the spectra consist of two components (SiC_{Bi} and SiC_{ZLG}), indicating slight de-intercalation owing to the preparation of the precursor phase from the α phase by annealing (see also Methods section). The components are separated because of a different surface band bending underneath intercalated and non-intercalated regions. The degree of intercalation is about 70–80 %, with the non-intercalated part of the sample surface being covered by a ZLG. Hence, in the C $1s$ spectrum, not only the signal (G_{Bi}) stemming from the graphene layer on top of the precursor phase but also the components S1 and S2 attributed to the ZLG are identified.

After H-intercalation we observe significant changes in the Si $2p$ and C $1s$ spectra. The graphene C $1s$ peak now consists of two components, while the ZLG components have vanished. This is due to the ZLG being converted into H-intercalated QFG, which gives rise for a G_H component close to the G_{Bi} peak. Thus, beside the graphene-protected bismuthene (originating from the precursor domains) we now find H-intercalated QFG regions on the surface. The C $1s$ peak positions indicate slight n- and p-type doping of the respective graphene layers, in agreement with the ARPES results, see main text. The Si $2p$ spectra can now be fitted by only one single component, which indicates that both of these domains exhibit a comparable surface band bending.

5 Stability in air and reversible switching of the QSHI state

As discussed in the main text, a key feature of the system presented in this work is the intrinsic protection of the quantum spin Hall insulator bismuthene against environmental degradation provided by the graphene sheet above (and the substrate below) the Bi layer. In order to check the efficiency of this protection, we exposed a bismuthene sample to air for 24 h. The upper overview spectrum shown in Figure S.6(a) was recorded from a bismuthene sample (Bi+H) immediately after preparation,

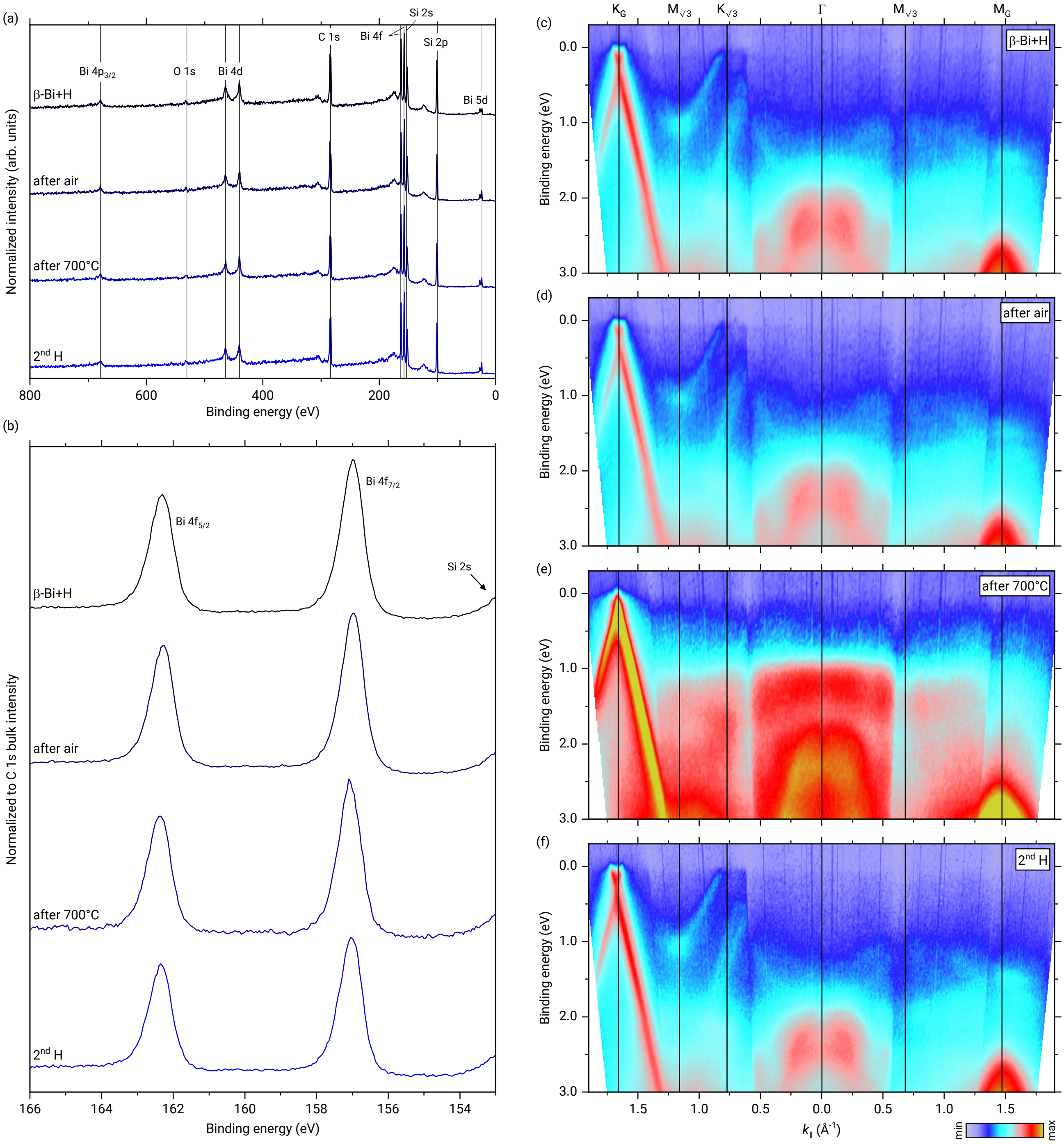

Figure S.6: PES data of a bismuthene sample directly after preparation (Bi+H), after exposure to air for 24 h (after air), after subsequent annealing at 700 °C (after 700 °C), and after the 2nd hydrogenation. (a) XP survey spectra, indicating that no significant oxidation takes place. (b) Bi $4f$ core level spectra (normalized to the respective C $1s$ bulk peak). (c-f) ARPES data taken along the $K_G - \Gamma - M_G$ direction of graphene, indicating that the band structure of graphene is preserved during exposure to air, reversed to the precursor phase characteristics upon annealing to 700 °C, and completely restored upon a second hydrogenation.

revealing that no significant amount of oxygen is present on the sample. After exposure to air, this has not changed, as demonstrated by the spectrum shown in the center of the panel labeled "after air", confirming that no oxidation took place at the surface and in the heterolayer system. This conclusion is confirmed by the XP spectra of the Bi $4f$ core level shown in Figure S.6(b). The shape of the Bi $4f$ core level spectra recorded before and after exposure to air (Bi+H and after air) shown in the upper part and the center of the panel are basically unchanged, in particular, no additional shifted peaks can be observed that could be attributed to oxidized bismuth. For reference, the binding energy of the Bi $4f_{7/2}$ orbital in Bi_2O_3 is 158.7 eV [3].

However, the proof for the bismuthene layer being stable under ambient conditions is provided by ARPES. The photoemission maps presented in Figure S.6(c) and (d) depict the band structures of the sample immediately after preparation and after exposure to air for 24 h, respectively. Also here, no significant differences between the two spectra can be observed, confirming the stability of the graphene / bismuthene heterostructure in air.

Furthermore, we have performed experiments to investigate the reversible switching between precursor and bismuthene. The limiting factor for the thermal stability of bismuthene is expected to be the H-saturation of the Si dangling bonds, i.e., the Si-H bonds located in the center of the bismuthene honeycomb. Previous studies on H-intercalated QFG reported de-intercalation, i.e., the breaking of the Si-H bond, at annealing temperatures of approximately 700 °C [4]. A similar stability is therefore expected for the present system. The curves in Figure S.6(a) and (b) labeled after 700 °C display the survey XP spectrum and the Bi $4f$ core level spectrum, respectively, after annealing at 700 °C. No significant changes are observed when comparing the two spectra to those before annealing. In particular, the intensities of the Bi $4f$ spectra (which are normalized to the C $1s$ bulk intensity), reveal that the overall Bi coverage remains unchanged.

The ARPES data obtained for the sample after annealing is shown in Figure S.6(e). In comparison with Figure 1b of the main text, it clearly demonstrates that, due to the annealing, the band structure has reverted to that of the precursor phase (before hydrogenation). In particular, the characteristic bismuthene Dirac bands have vanished, and the insulating state previously associated with the precursor phase has been reestablished. Furthermore, the doping of the graphene layer has changed from n-type to charge neutrality, consistent with the observations for the precursor phase. Thus, the QSHI state of bismuthene can be switched off and transformed back into the precursor by annealing at sufficiently high temperatures at which the Si-H bond breaks and the hydrogen desorbes.

To complete the switching cycle, we repeated the hydrogenation process with the dehydrogenated sample to restore the QSHI state. The XP survey and Bi $4f_{7/2}$ spectra after the second hydrogenation of this sample are shown in Figure S.6(a) and (b), labeled "2nd H". No obvious changes can be seen compared to the first hydrogenation, which especially emphasizes that the amount of Bi remains constant throughout the cycle. More importantly, the corresponding energy-momentum map in Figure S.6(f) shows all the features of bismuthene again. The data are basically identical to those recorded after the first hydrogenation (Figure S.6(c)): (i) The fully occupied surface state attributed to the precursor has disappeared. (ii) The Dirac-like bands of bismuthene appeared again throughout the entire Brillouin zone. (iii) The graphene doping changed back to n-type. In all the data we see no signs for degradation of the bismuthene. Note that also after the second hydrogenation the p-doped graphene band appears again, indicating the presence of solely H-intercalated regions, which were transformed into ZLG in the dehydrogenation process and became reintercalated during the second hydrogenation process.

We conclude that the QSHI state of bismuthene can be reversibly switched off and back on after the initial preparation. Due to the complexity of the hydrogenation process, we have performed only two complete switching cycles, but detected no indications for any sample degradation. Thus, we propose that multiple switching is possible at least several times without a significant loss in sample quality.

6 Hydrogenation of the α phase

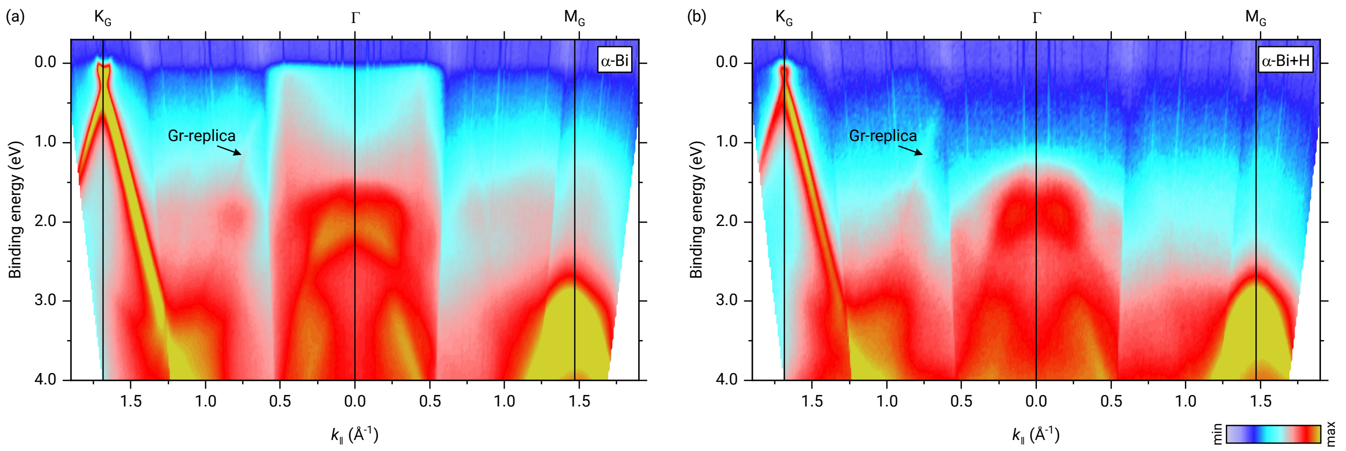

Figure S.7: ARPES data of a Bi α phase (α-Bi) along $K_G - \Gamma - M_G$ direction of graphene before (a) and after (b) hydrogen treatment. Both photoemission maps show no indications for the formation of bismuthene.

Figure S.7 shows photoemission maps of the Bi α phase before (a) and after (b) hydrogen treatment. No significant changes of the electronic structure are observed. In particular, no bismuthene bands can be observed in Figure S.7 (b), but only the valence band maximum of the SiC substrate at Γ and the graphene π-bands with their characteristic linear dispersion at K_G together with a π-band replica.

We conclude that beside the saturation with hydrogen, also the structural $(\sqrt{3} \times \sqrt{3})$ template of the Bi β structure is essential for the formation of bismuthene.

References

[1] D. M. Pakdehi, P. Schädlich, T. T. N. Nguyen, A. A. Zakharov, S. Wundrack, E. Najafidehaghani, F. Speck, K. Pierz, T. Seyller, C. Tegenkamp, and H. W. Schumacher: Silicon Carbide Stacking-Order-Induced Doping Variation in Epitaxial Graphene. Adv. Funct. Mater. **30**(45), 2004695 (2020).

[2] J. B. Pendry: Reliability factors for LEED calculations. Journal of Physics C: Solid State Physics **13**(5), 937 (1980).

[3] L. Z. Zhao and J. B. Zhang: Bi and O valences in Ba-K-Bi-O, Ba-K-M-Bi-O (M = Rb, La, Eu, In, Tland Pb) and the related compounds. Solid State Commun. **90**(11), 709 (1994).

[4] C. Riedl, C. Coletti, T. Iwasaki, A. A. Zakharov, and U. Starke: Quasi-Free-Standing Epitaxial Graphene on SiC Obtained by Hydrogen Intercalation. Phys. Rev. Lett. **103**(24), 246804 (2009).
